# Evaluation of the mesenteric arterial vasculature by computed tomography angiography and its implications for colorectal cancer surgery

**DOI:** 10.1590/0100-3984.2023.0099

**Published:** 2024-05-07

**Authors:** Marcelo Castro, Javiera Cornejo, Mauricio Acuña, Laura Naim, José Vía Dorado, Lía Rodríguez, Sebastián Aguirre, David Herquiñigo

**Affiliations:** 1 Department of Radiology, Division of Abdominal Imaging, INDISA Clinic, Santiago, Chile; 2 Universidad Andrés Bello School of Medicine, Santiago, Chile

**Keywords:** Mesenteric artery, inferior, Computed tomography angiography, Colorectal neoplasms, Laparoscopy, Blood vessels/anatomy & histology, Anatomic variation, Artéria mesentérica inferior, Angiografia por tomografia computadorizada, Neoplasias colorretais, Laparoscopia, Vasos sanguíneos/anatomia & histologia, Variação anatômica

## Abstract

**Objective:**

To determine the branching patterns of the inferior mesenteric artery (IMA) and to describe the clinical applicability of computed tomography (CT) angiography in the evaluation of these vessels to facilitate the planning of colorectal cancer surgery.

**Materials and Methods:**

We included 100 patients who underwent CT angiography of the abdomen and pelvis. The branching patterns of the IMA were examined and classified as type 1 (bifurcated), including 1A (sigmoid and left colic arteries arising from a common trunk), 1B (sigmoid and superior rectal arteries arising from a common trunk) and 1C (sigmoid arteries arising from both trunks); type 2 (trifurcated); and type 3 (no left colic branch).

**Results:**

Among the 100 patients evaluated, we found the variant to be type 1A in 9%, type 1B in 47%, type 1C in 24%, type 2 in 16%, and type 3 in 4%.

**Conclusion:**

Preoperative CT angiography for evaluating the IMA branching pattern could inform decisions regarding the surgical approach to colorectal cancer.

## Abstract (pt)

**Objetivo:**

Determinar os padrões de ramificação da artéria mesentérica inferior (AMI) e descrever a aplicabilidade clínica da angiografia por tomografia computadorizada na avaliação desses vasos na elaboração das estratégias pré-operatórias de cirurgia de câncer colorretal.

**Materiais e Métodos:**

Foram incluídos 100 pacientes submetidos a angiografia por tomografia computadorizada abdominal e pélvica. Os padrões de ramificação da AMI foram examinados e classificados como tipo 1 (bifurcado), incluindo 1A (artérias sigmoide e cólica esquerda originando-se de um tronco comum), 1B (artérias sigmoide e retal superior originando-se de um tronco comum) e 1C (artérias sigmoide originando-se de ambos os troncos); tipo 2 (trifurcado); e tipo 3 (sem ramo cólico esquerdo).

**Resultados:**

Do total de participantes incluídos no estudo, a variante do tipo 1A foi observada em 9%, a do tipo 1B em 47%, e a do tipo 1C em 24%. Com relação à variante tipo 2, esta foi observada em 16% dos pacientes, e a do tipo 3, em 4% dos casos.

Conclusão O uso da angiografia por tomografia computadorizada pré-operatória para avaliar o padrão de ramificação da AMI pode ajudar a escolher a abordagem cirúrgica no câncer colorretal.

## INTRODUCTION

Worldwide, colorectal cancer is the third leading type of cancer and the fourth leading cause of cancer-related death^(1)^. During the last decade, the minimally invasive approach has been widely incorporated in daily practice. Although numerous studies have shown that laparoscopic resection results in faster recovery with similar oncological outcomes in comparison with an open approach, because of the narrow field of view and lack of tactile sensation during laparoscopy, the vascular anatomical structures tend to be misidentified and can be injured as a result^(2,3)^.

The vessels supplying the colon and rectum have several anatomical variants. The inferior mesenteric artery (IMA) is the predominant supply vessel for the left colon and rectum. The branching pattern of this artery also varies among individuals, and there are few anatomical, angiographic, or surgical studies that support what is traditionally taught as normal in anatomy texts^(3)^. Although the origin of the IMA is fairly constant, the branching pattern of its arteries-the left colic artery (LCA), sigmoid trunk, and superior rectal artery-is highly variability in terms of the origin and number of those branches, as well as the presence or absence of secondary branches^(4,5)^. Identifying these variations is essential to devising preoperative strategies, in order to determine the arterial branching pattern, the knowledge of which is quite helpful to surgeons, who must make decisions regarding vessel resection and lymph node dissection, to avoid anastomotic complications^(6-8)^.

There are multiple classifications for the IMA system, which is why McSweeney et al.^(5)^ proposed a step-by-step system that minimizes the number of divisions and simplifies the evaluation. The first step is determining whether the IMA bifurcates or trifurcates. A bifurcated IMA is classified as type 1, which is subdivided as follows: type 1A (if the sigmoid artery and LCA arise from a common trunk); type 1B (if the sigmoid artery and superior rectal artery arise from a common trunk); and type 1C (if sigmoid arteries arise from both trunks). A trifurcated IMA is classified as type 2. Finally, an IMA that has no LCA branch is classified as type 3, which is quite rare^(5)^.

Computed tomography (CT) angiography has become the method of choice for assessing vascular anatomy because of its high sensitivity and specificity, as well as because it is minimally invasive and its acquisition times are short^(9)^. Through visual tracking, mesenteric arteries can be traced to their terminal branches during CT angiography, making it the method of choice for the preoperative evaluation of vascular anatomy^(4,10)^. The aim of the present study was to determine the branching patterns of the IMA and to describe the clinical applicability of CT angiography in the evaluation of the vessels, to facilitate the planning of colorectal cancer surgery.

## MATERIALS AND METHODS

This was a retrospective descriptive observational study involving 100 patients who underwent CT angiography of the abdomen and pelvis between 2019 and 2022 at a hospital in Santiago, Chile. Patients with a history of abdominal surgery, thrombosis of the IMA, or endovascular procedure were excluded, as were those in whom the CT angiography was of poor diagnostic quality. This study was approved by the scientific ethics committee of our institution. Because of the retrospective nature of the study, the requirement for informed consent was waived.

To calculate the sample size, we considered as a reference the prevalence rates previously reported for the variant types established in the IMA classification system proposed by McSweeney et al.^(5)^. The event of interest was the anatomical variant with the lowest frequency of presentation, with a proportion of 2%, a confidence level of 95%, and an accuracy of 3%, adjusted to losses of 15%, which resulted in a sample size of 98 participants, a number that was rounded to 100 to facilitate the statistical analysis. Patients were enrolled in reverse chronological order until the total was achieved.

All CT angiography examinations were performed in a 128-slice scanner (IQon spectral CT; Philips Medical Systems, Best, The Netherlands) or in a 64-slice scanner (Brilliance; Philips Medical Systems). The tube voltage was 120 kVp, and the tube current was 66-200 mAs. Patients fasted for at least four hours prior to the examination, which was acquired in the supine position. An average of 100 mL of iodinated contrast was injected into median cubital vein, at a rate of 4.5 mL/s. The bolus tracking method was used in order to decide the scan timing. Acquisition of the arterial phase was automatically initiated when the density at the level of the thoracic aorta trunk reached 130 HU. The slice thickness was 1 mm, reconstructing into 0.5-mm images. Image processing analysis was performed using a three-dimensional (3D) volume rendering technique.

**Figure 1:**
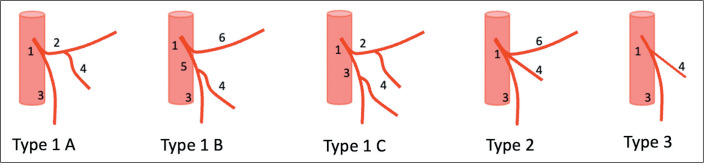
McSweeney et al.^(5)^ classification system for the IMA branching pattern. IMA (1); colosigmoid artery (2); superior rectal artery (3); sigmoid trunk (4); rectosigmoid artery (5); and LCA (6).

Two radiologists, one with 2 years of experience and another with 27 years of experience, processed the images and identified the blood vessels using conventional images and 3D reconstructions. Working in consensus, they classified the vascular anatomy of each participant using the McSweeney et al.^(5)^ IMA classification system (Figure 1).

Statistical analyses were performed with Stata software, version 17.0 (Stata Corp LP, College Station, TX, USA). Descriptive statistics were used in order to summarize the demographic characteristics of the participants. The frequency of the IMA variants was expressed as absolute values and percentages. Student’s t-tests were used to analyze differences between men and women in terms of the anatomical variations of the IMA.

## RESULTS

Among the 100 patients evaluated, the median age was 53.3 years, and 53% were men. In accordance with the McSweeney et al. IMA classification system^(5)^, we identified type 1A in 9% of the patients, type 1B in 47%, type 1C in 24%, type 2 in 16%, and type 3 in 4%. As can be seen in Table 1, there were no statistically significant differences between men and women in terms of the frequency of the anatomical variations of the IMA (*p* = 0.04).

**Table 1:** Absolute frequencies of the branching patterns of the IMA, by sex and age.

			IMA variant type				
	Age (years)	1A	IB	1C	2	3	Total
Sex	Mean	n	n	n	n	n	n
Male	56.6	5	31	10	5	2	53
Female	49.7	4	16	14	11	2	47
Total	53.4	9	47	24	16	4	100

## DISCUSSION

In colorectal cancer surgery, the standard procedure includes removal of the tumor, radical resection of the colonic mesentery, and ligation of the IMA. There are two options for IMA ligation: high ligation, above the LCA branch, and low ligation, with preservation of the LCA^(11)^. Defining the level of ligation of the IMA ensures the best blood supply while avoiding mesenteric tension upon the anastomosis, thus avoiding anastomotic dehiscence, which is one of the most feared complications because it is associated with significant morbidity and mortality^(12)^.

Many surgeons prefer to perform high IMA ligation to facilitate a complete lymphadenectomy and better mesenteric mobilization while avoiding tension on the anastomosis, although the use of that technique can result in insufficient blood supply to the colon. Therefore, some authors have stated the use of low IMA ligation, with preservation of the LCA, could be beneficial, given that it can provide better blood supply to the anastomosis, although it is a more technically complex procedure, and that preoperative assessment of the IMA branching pattern could facilitate the decision of which technique to use^(13,14)^.

There is no consensus among surgeons regarding the IMA ligation level. However, given the increasing use of laparoscopy for this kind of surgery, together with the impaired visibility during laparoscopic procedures, studies on this topic should consider radiological evaluation, preoperatively to characterize the vascular anatomy for the surgical planning and postoperatively to confirm the IMA ligation level and irrigation of the anastomosis. In addition, understanding of the anatomy of the IMA could facilitate the individualization of the surgical planning and of the postoperative management.

Recent advances in CT have allowed excellent visualization of the vascular anatomy. Various studies have shown that vascular anatomical relationships in the root of the mesentery can be seen on preoperative CT and have demonstrated the effectiveness of identifying mesenteric anatomical variants preoperatively, with high specificity, sensitivity, accuracy, and reliability in comparison with identifying those variants intraoperatively^(15-17)^.

**Figure 2:**
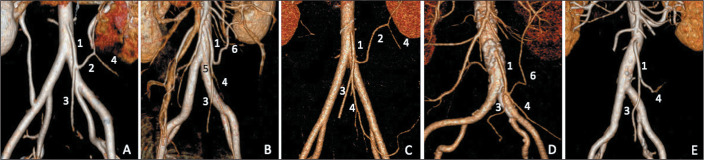
3D reconstruction of the IMA and its branching patterns. A: Type 1A. B: Type 1B. C: Type 1C. D: Type 2. E: Type 3. IMA (1); colosigmoid artery (2); superior rectal artery (3); sigmoid trunk (4); rectosigmoid artery (5); and LCA (6).

The anatomy of the IMA is highly variable, and there is no universal classification system. However, in 2020, McSweeney et al.^(5)^ carried out a systematic review that included 44 articles describing the anatomy of the IMA and proposed a classification system that involves a series of key steps. The system is easily and quickly applied, can be used by the radiologist in the report provided to the surgeon for preoperative planning, and provides a standardized categorization. Therefore, we employed this classification system as a reference when determining the prevalence of the various anatomical variants of the IMA branching pattern, using conventional CT angiography images and 3D reconstructions. In our patient sample, the IMA presented significant variability. We found that the most common branching pattern was type 1B, followed by type 1C, type 2, and type 3, with no statistical differences related to sex or age (Figure 2). Although McSweeney et al.^(5)^ evaluated the prevalence of the different IMA variants according to that reported in other studies, these are not comparable data, given that those studies used different classification systems and did not report frequencies for all subtypes, thus precluding comparisons with other populations. That is why it is important to use a standardized classification system that can be used in order to compare prevalence between and among future populations^(5)^.

Our study has some limitations. The sample was drawn from a single center and is therefore not necessarily representative. In addition, the sampling was non-probabilistic, which can also influence the results. Furthermore, the vascular anatomy of the IMA was measured in healthy patients and not in patients with colorectal cancer, in whom anatomical variations could have different implications for the reconstruction of images.

Although radiologists have developed the visual capacity and geometric skill to build 3D models of the anatomy from two-dimensional images, the acquisition of actual 3D images could help them better detect the branching pattern of the IMA preoperatively^(18)^. This is a widely available tool that can precisely predict the difficulties of surgery and facilitate the development of individualized operational strategies, thus decreasing operative time and reducing the risk of anastomotic ischemia.

## CONCLUSION

Preoperative CT angiography for evaluating the IMA branching pattern could inform decisions regarding the surgical approach to colorectal cancer.
